# EndoE from *Enterococcus faecalis* Hydrolyzes the Glycans of the Biofilm Inhibiting Protein Lactoferrin and Mediates Growth

**DOI:** 10.1371/journal.pone.0091035

**Published:** 2014-03-07

**Authors:** Julia Garbe, Jonathan Sjögren, Eoin F. J. Cosgrave, Weston B. Struwe, Marta Bober, Anders I. Olin, Pauline M. Rudd, Mattias Collin

**Affiliations:** 1 Department of Clinical Sciences, Division of Infection Medicine, Lund University, Lund, Sweden; 2 Dublin Oxford Glycobiology Group, National Institute for Bioprocessing Research & Training (NIBRT), Mount Merrion, Blackrock, Co. Dublin, Ireland; Centre National de la Recherche Scientifique, Aix-Marseille Université, France

## Abstract

Glycosidases are widespread among bacteria. The opportunistic human pathogen *Enterococcus faecalis* encodes several putative glycosidases but little is known about their functions. The identified endo-β-*N*-acetylglucosaminidase EndoE has activity on the N-linked glycans of the human immunoglobulin G (IgG). In this report we identified the human glycoprotein lactoferrin (hLF) as a new substrate for EndoE. Hydrolysis of the N-glycans from hLF was investigated using lectin blot, UHPLC and mass spectrometry, showing that EndoE releases major glycoforms from this protein. hLF was shown to inhibit biofilm formation of *E. faecalis in vitro*. Glycans of hLF influence the binding to *E. faecalis,* and EndoE-hydrolyzed hLF inhibits biofilm formation to lesser extent than intact hLF indicating that EndoE prevents the inhibition of biofilm. In addition, hLF binds to a surface-associated enolase of *E. faecalis*. Culture experiments showed that the activity of EndoE enables *E. faecalis* to use the glycans derived from lactoferrin as a carbon source indicating that they could be used as nutrients *in vivo* when no other preferred carbon source is available. This report adds important information about the enzymatic activity of EndoE from the commensal and opportunist *E. faecalis*. The activity on the human glycoprotein hLF, and the functional consequences with reduced inhibition of biofilm formation highlights both innate immunity functions of hLF and a bacterial mechanism to evade this innate immunity function. Taken together, our results underline the importance of glycans in the interplay between bacteria and the human host, with possible implications for both commensalism and opportunism.

## Introduction

*Enterococcus faecalis* is a well-known member of the microbial consortium in the human gastrointestinal tract and the oral cavity [1], [2], but it is also known to cause infectious endocarditis [3], urinary tract infections [4], bacteremia as well as sepsis [5]. The treatment of enterococcal infections is difficult due to the increasing antibiotic resistance which can be transferred among *E. faecalis* strains via mobile genetic elements [1], [6]. It has also been shown that *E. faecalis* is able to adhere to and form biofilms on different biomaterials and medical devices suggesting an involvement of biofilms in the process of infection [7]–[11].

Many human pathogens have evolved intricate mechanisms to survive in the human body. Escaping the immune system and gaining nutrients are challenging obstacles for pathogenic bacteria. Secreted proteins with glycosidase activity have been identified in different bacteria such as *Streptococcus pyogenes* [12], [13], *Streptococcus oralis* [14] and *Capnocytophaga canimorsus* [15]. Glycosidases from pathogens can target different glycoproteins of the host, for example, proteins of the immune system, thereby changing the function of the protein and help the intruding bacteria to escape the immune response [16]–[19]. Prominent examples of pathogenic glycosidases are EndoS from *S. pyogenes* and the pneumococcal enzymes NanA, BgaA and StrH. EndoS is able to cleave the N-linked glycans from IgG and thereby inhibit the immunoglobulin-mediated opsonophagocytosis, which increases the survival of *S. pyogenes* in blood [20]. The three exoglycosidases NanA, BgaA and StrH from *Streptococcus pneumoniae* are responsible for the sequential deglycosylation of human glycoproteins and play a major role in evasion of opsonophagocytosis, adherence to epithelial surfaces and nutrient acquisition [17]. Bacterial hydrolysis of host glycoproteins can have direct modulating activity immune functions as exemplified by endoglycosidase with activity on human antibodies. In addition to this, it is clear that several bacteria, such as oral streptococci, liberate carbohydrates that can be used as nutrients during colonization and infection [14], [21].

The endoglycosidase EndoE from *E. faecalis* was identified due to similarities to the well investigated endoglycosidase EndoS from *S. pyogenes* [22]. EndoE has a unique feature, namely, it combines two enzymatic domains with different glycosyl hydrolase activities. The α domain of EndoE contains a family 18 glycosyl hydrolase (GH18) motif while the β domain contains a family 20 glycosyl hydrolase (GH20) motif and is able to release the glycans from the immunoglobulin IgG [22].

Many of the proteins involved in adaptive and innate immunity are glycosylated. The carbohydrates are important for the stability and also for recognition [23]. The non-heme iron binding protein lactotransferrin (lactoferrin, hLf) belongs to the family of transferrins that are involved in the regulation of iron homeostasis [24]. This protein has a mass of 80 kDa, is present at concentrations of up to 7 mg/ml in milk but can also be found on mucosal surfaces, for example in the gastrointestinal tract or the respiratory tract, at lower concentrations around 2 mg/ml [25]. More recently, hLF has been described to have multiple biological functions in blood and mucosal surfaces and is considered as a component of the innate immune system with antimicrobial activity [26]–[29]. The antimicrobial activity of this protein has been attributed to deprivation of essential iron [30], outer membrane damage in Gram-negative bacteria [31], and recently, the inhibition of bacterial H^+^-ATPase [26]. Moreover, it has been described that hLF is able to inhibit biofilm formation of bacteria like *Pseudomonas aeruginosa, Streptococcus mutans*, *Porphyromonas gingivalis* and *Prevotella intermedia* [32]–[34]. Human hLF from milk contains three putative N-glycosylation sites (Asn138, Asn479 and Asn624), of which only two sites (Asn138 and Asn479) are linked to highly branched, highly sialylated and highly fucosylated complex type N-glycans [35]. Not much is known about the role of the glycans, but it has been described that de-glycosylated hLF has identical affinity to iron and bacterial lipopolysaccharide [36].

In this study, we hypothesized that the endoglycosidase EndoE from *E. faecalis* has activity on human glycoproteins and that this activity might help *E. faecalis* to persist and/or survive in the human body. We showed that EndoE cleaves complex type N-linked glycans from hLF, which leads to a reduction of the biofilm inhibiting properties of this protein. Removal of the glycans by EndoE restored biofilm formation of *E. faecalis*, showing a new mechanism for *E. faecalis* to interfere with a possible defense mechanism of the human body. In addition, the released glycans can be metabolized by *E. faecalis* and could support nutrient acquisition.

## Results

### EndoE Binds to and has Glycosidase Activity on Human Lactoferrin from Milk

It has been described previously that the β-*N*-acetylglucosaminidase EndoE has activity on the glycans of human IgG and on the glycans of the model glycoprotein RNaseB [22]. We identified a new substrate for EndoE, the human glycoprotein lactoferrin (hLF), that contains two complex type N-glycans [35].

**Figure 1:**
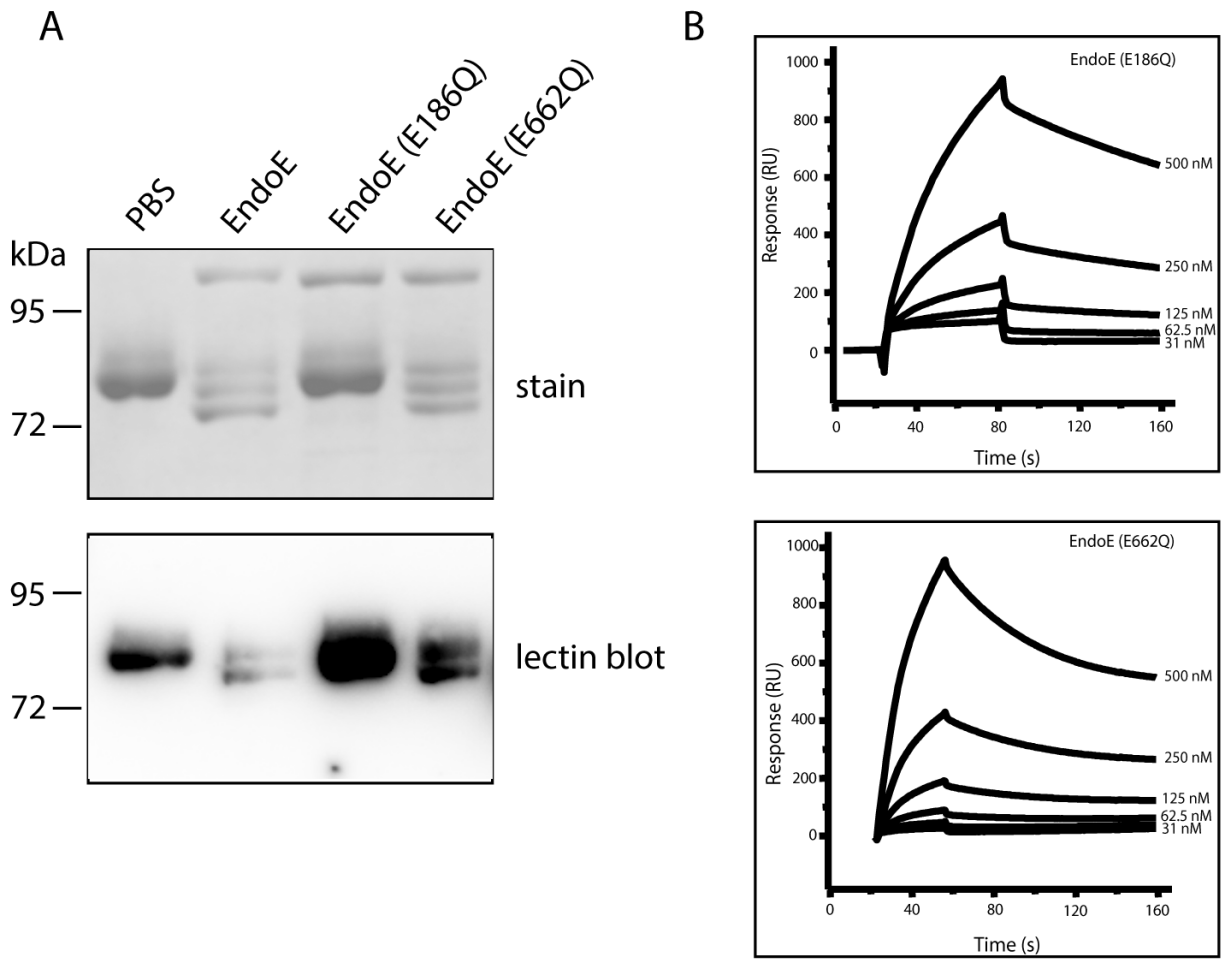
Activity on and binding of EndoE to human lactoferrin. A. Human lactoferrin (hLF) was incubated with EndoE, EndoE(E186Q) and EndoE(E662Q), separated on 10% SDS-PAGE and stained with Coomassie (upper panel), or electro-blotted onto PVDF membranes and was analyzed with ConA lectin (lower panel). Incubation of hLF with PBS was used as a negative control. B. Plasmon surface resonance assay to analyze binding of EndoE to hLF. The plots show binding of EndoE(E186Q) and EndoE(E662Q) to hLF.

To investigate the glycan hydrolyzing activity of EndoE, recombinant enzyme or PBS as control was incubated with hLF from milk and analyzed with SDS-PAGE (Fig. 1 A, upper panel) and Concanavalin A (ConA) lectin blot (Fig. 1 A, lower panel), detecting α-mannosidic structures of the N-linked glycan. EndoE was found to have glycosidase activity on hLF as indicated by a shift of the native glycoprotein into three bands compared to the control seen as one band at 80 kDa on the SDS-PAGE gel. The lectin blot analysis showed that the lowest band in the SDS gel corresponds to de-glycosylated hLF since no signal was obtained with the ConA lectin, indicated by the lack of the last band in the lectin blot. Since hLF contains two N-linked glycans, the presented results indicate that lactoferrin occurs in three protein forms after EndoE treatment: fully glycosylated hLF (hLF with the highest molecular weight, similar to the control), hLF with partially cleaved N-linked glycans and de-glycosylated hLF. However, the possible presence of one or two remaining *N*-acetylglucosamines (GlcNAc) and a α1–6-linked fucose on the protein backbone as well as the nature of the partially cleaved N-linked glycans cannot be verified with this method but will be further described below. It has been described previously that EndoE has two domains with different enzymatic activity [22]. To investigate which domain is responsible for the glycan hydrolysis on hLF, we incubated the glycoprotein with site-directed mutagenized EndoE. As described previously, two glutamic acid residues (Glu186 and Glu662), which are important for the activity of the α domain and β domain, respectively, were exchanged to glutamine, leading to an EndoE with active α domain but inactive β domain (EndoE(E662Q)) and an EndoE with active β domain but inactive α domain (EndoE(E186Q)) [22]. Fig. 1A shows that the α domain of EndoE with family 18 glycosyl hydrolase activity is responsible for the hydrolysis of hLF since the mutated EndoE(E186Q) lost its activity on hLF.

To verify the physical interaction between EndoE and lactoferrin, surface plasmon resonance analysis with EndoE(E186Q) and EndoE(E662Q) was performed (Fig. 1B). This revealed that both, EndoE(E186Q) with inactive α domain and EndoE(E662Q) with inactive β domain, interact with hLF. The K_D_ value for EndoE(E662Q) was calculated to be 3.7 nM whereas the K_D_ value for EndoE(E186Q) was calculated to be 44 nM, indicating a stronger interaction of hLF with EndoE(E662Q) than EndoE(E186Q) of which the latter variant shows no activity on hLF in the lectin blot analysis (see above). No interaction between wild type EndoE and hLF could be detected using this method (data not shown). This is most likely due to a transient interaction between the active endoglycosidase and the substrate glycoprotein as has been shown for the related enzyme EndoS [16].

### EndoE Releases Major Glycoforms from Human Lactoferrin

**Figure 2:**
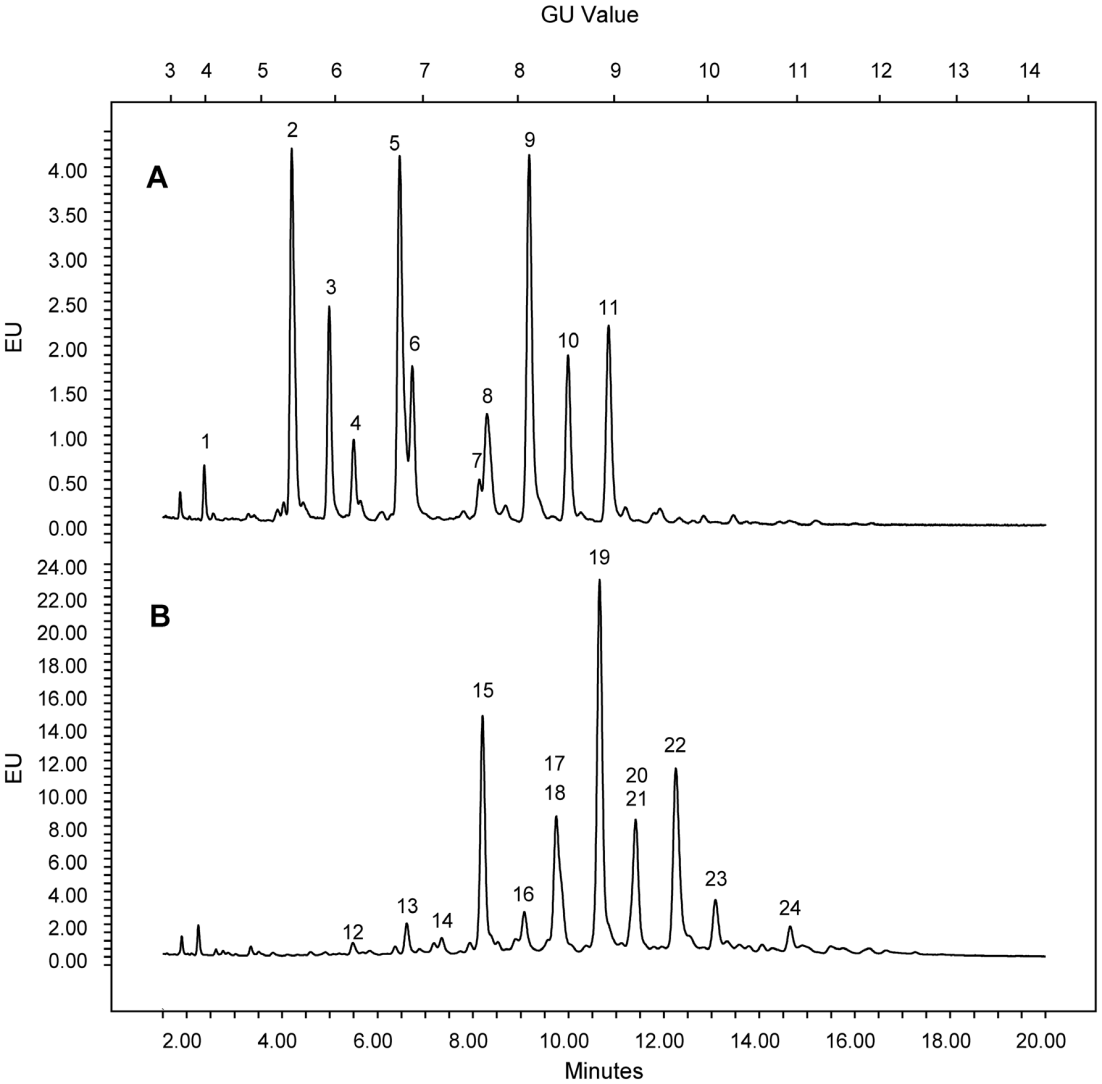
Glycan analysis of lactoferrin. Hydrophilic interaction liquid chromatography (HILIC)-fluorescence chromatogram of 2-AB labeled glycans released from human lactoferrin by the endoglycosidase EndoE (A) and the endoglycosidase PNGaseF, respectively (B). Identified glycans are separated into peaks. The numbers correspond to the glycan structures depicted in Figure 3 and 4.

As described above, EndoE is able to hydrolyze glycans from hLF (Fig. 1A). Although we detected de-glycosylated hLF, we also detected fully glycosylated hLF and hLF with partially cleaved glycans according to the SDS-PAGE gel and ConA lectin blot. Longer incubation did not change the shifts to only de-glycosylated hLF (data not shown), indicating that EndoE might not have activity on all occurring N-glycan structures of hLF. The glycosylation profile of hLF from milk was recently investigated, indicating the presence of 17 different, complex-type N-glycan structures attached to hLF on two sites: Asn138 and Asn479 [35]. Moreover, it has also been described that the glycans at Asn138 were more complex, more branched and more fucosylated [35]. To investigate what glycans were hydrolyzed by EndoE, the N-glycans from lactoferrin were released with either PNGaseF or EndoE and comparatively analyzed using UHPLC (Ultra high performance liquid chromatography) and LC-MS (liquid chromatography-mass spectrometry) (Fig. 2). PNGaseF (Peptide-N-Glycosidase F from *Elizabethkingia meningoseptica*) is an amidase that is widely used in glycan analysis of N-linked glycans as a control enzyme that hydrolyzes the bond between the protein backbone and the first GlcNAc of the glycan.

**Figure 3:**
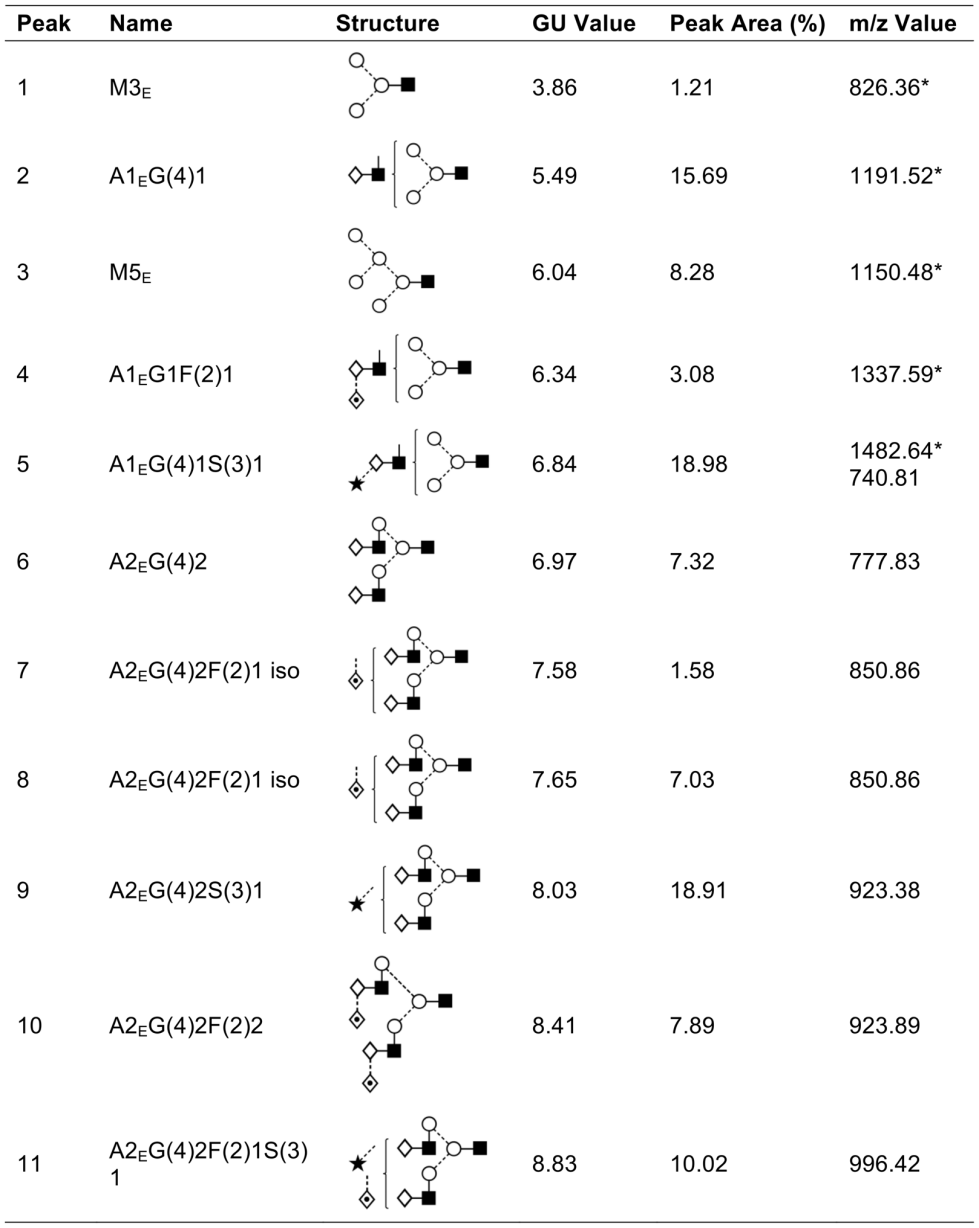
EndoE released N-glycans identified from human lactoferrin. The depicted glycan structure is based on the Oxford glycan nomenclature [56]. Glycans were detected as [M-H] and [M-2H]^2−^ ions. * denotes single charged ions. Glycan names denoted with a subscript E refer to glycans released using EndoE. GU values were generated as previously described [54]. In situations where chromatographic peaks containing multiple structures, the associated peak area was divided equally among the structures for simplicity.

**Figure 4:**
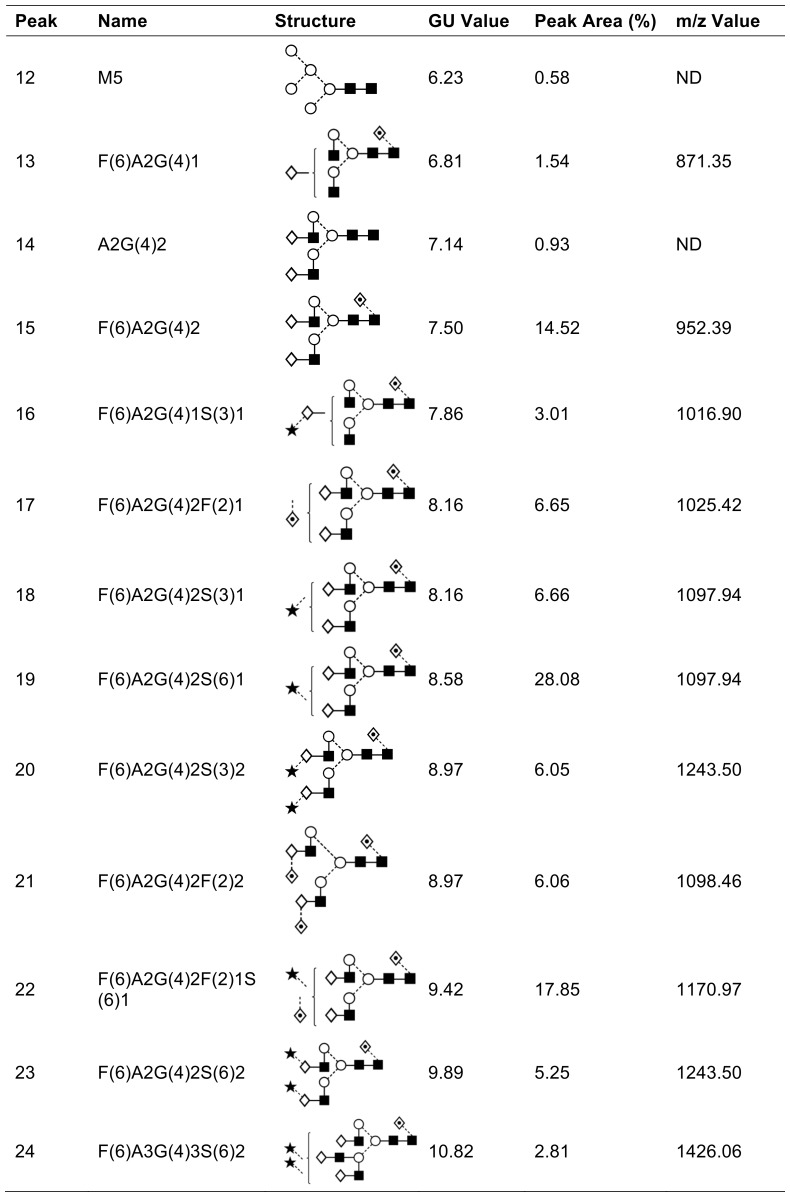
PNGaseF released N-glycans identified from human lactoferrin. The depicted glycan structure is based on the Oxford glycan nomenclature [56]. Glycans were detected as [M-2H]^2−^ ions. GU values were generated as previously described [54]. In situations where chromatographic peaks containing multiple structures, the associated peak area was divided equally among the structures for simplicity.

It has previously been shown that EndoE cleaves between the two GlcNAc residues in the chitobiose core structure of IgG N-glycan [22], and this activity was also confirmed on hLF (Figure 3). Most glycoforms of hLF that were identified in the analysis released by PNGaseF could also be detected in the glycan profile of hLF incubated with EndoE, as indicated by the peaks in the chromatogram (Peaks 3, 6, 7, 8, 9, 10). The peaks corresponding to the glycans released by PNGaseF are shifted by roughly 0.5 GU (glucose unit) since it releases both GlcNAcs from the protein whereas EndoE cleaves between the two core GlcNAcs. The structures from the identified released glycans are shown in Figure 3 and 4. Interestingly, peaks 2, 4 and 5 from the chromatogram correspond to glycans with single antennary structures that were released by EndoE but were not found among the glycans released by PNGaseF. However, these glycans could be the products of the glycans 14/15, 21 and 20 respectively, but further processed by glycosidase activity between the mannose and the GlcNAc. Also, glycans containing sialic acid α1–6-linked to galactose released by PNGaseF (19, 22, 23 and 24) were not found in the glycan profile of EndoE. This indicates that EndoE might not be able to cleave the glycans containing sialic acids, which could be an explanation for the detection of hLF with partial deglycosylation in the lectin blot analysis.

### Lactoferrin Inhibition of Biofilm Formation of *E. faecalis* is Glycan Dependent

**Figure 5:**
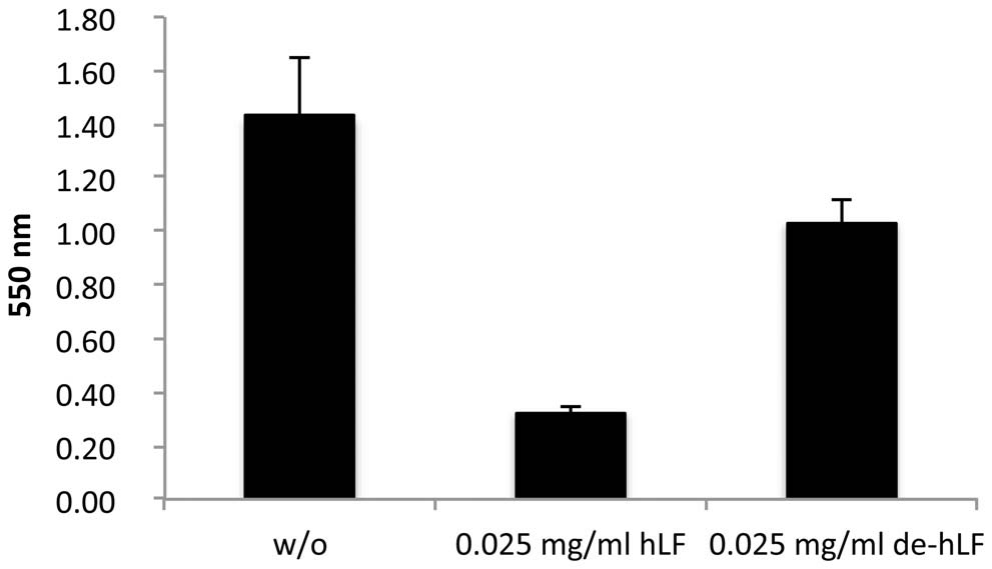
Influence of human lactoferrin on biofilm formation of *Enterococcus faecalis*. Biofilm formation of *E. faecalis* was measured using the crystal violet assay and is expressed as OD_550_. Human lactoferrin (hLF) was added either fully glycosylated (hLF) or deglycosylated (de-hLF) due to the treatment with EndoE. Error bars indicate the standard deviation from the mean of three independent experiments with three replicates. w/o: no hLF added.

The multifunctional protein hLF has been described to inhibit biofilm formation by several bacterial species. We investigated whether hLF affects the biofilm formation of *E. faecalis*. *E. faecalis* was grown in the presence of 0.025 mg/ml hLF in 96 well plates and attached cells were stained with crystal violet to examine biofilm formation (Fig. 5). In the presence of hLF, biofilm formation was inhibited by 80% compared to the control without hLF. Moreover, it has also been described that hLF has bactericidal effects on bacteria. To rule out any potential killing effect of hLF on biofilm formation, we measured the colony forming units (CFU) before staining with crystal violet and determined the amount of viable cells. No bactericidal effects for the used hLF concentration were observed in the experiment (data not shown). To rule out that the growth rate of *E. faecalis* is not affected by lactoferrin, which could cause decreased biofilm formation, we performed growth experiments in the presence of 0.1 mg/ml hLF but could not detect any effect on the growth rate of *E. faecalis* (data not shown). Next, we examined if the glycans of hLF could play a role in the inhibition of biofilm formation. Therefore, hLF was first treated with recombinant EndoE and purified. The purified deglycosylated hLF (de-hLF) was subsequently used for the biofilm assay as described above. Fig. 5 shows that the removal of glycans from lactoferrin restored biofilm formation of *E. faecalis*. However, the restoration of biofilm formation is not complete, which is probably due to the fact that EndoE cannot completely deglycosylate hLF (Fig. 1A).

### Lactoferrin Binding to the Surface Associated Enolase is Glycan Dependent

**Figure 6:**
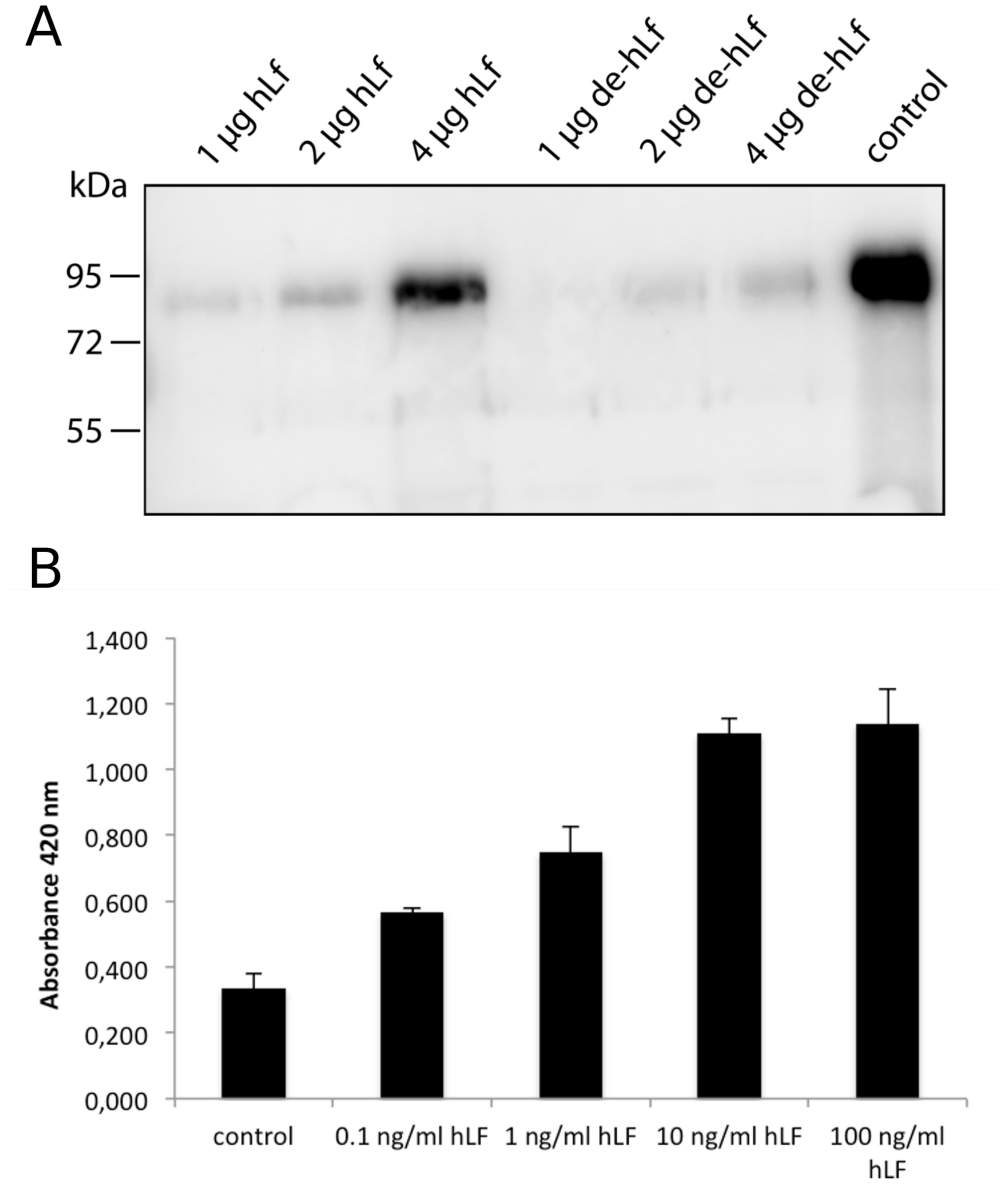
Binding of human lactoferrin to the surface of *Enterococcus faecalis* and recombinant enolase. A. Western blot analysis, using anti-lactoferrin antibodies, of human lactoferrin (hLF) bound to *E. faecalis* OG1. *E. faecalis* was incubated with indicated concentrations of hLF or EndoE treated hLF (de-hLF). *E. faecalis* cell extract was separated on 10% SDS-PAGE and electro-blotted onto PVDF membranes. Control: 1 µg hLF. B. Binding of different hLF concentrations to recombinant enolase immobilized to a microtiter plate. Anti-hLF antibodies were used to detect the binding of hLF to the enolase. Error bars indicate the standard deviation from the mean of three independent experiments.

Glycans are often important for the interaction or binding to other proteins or sugars. To identify the function of the biofilm inhibitory effect of hLF we investigated if hLF can bind to *E. faecalis* and whether it binds to a specific surface protein. The binding of hLF to the surface of *E. faecalis* could lead to a reduced adherence of *E. faecalis* to different surfaces and could explain the biofilm inhibitory effect described above. First, we incubated *E. faecalis* with hLF in different concentrations, washed the cells and detected cell bound hLF with Western blot analysis using anti-hLF antibodies (Fig. 6 A). We observed a concentration dependent binding of hLF to *E. faecalis* and investigated next if the binding is dependent on the glycans of hLF. Fig. 6 A shows that the binding of de-hLF to *E. faecalis* was strongly reduced compared to the fully glycosylated hLF. These results indicate that the binding to *E. faecalis* is glycan dependent and might be mediated by a specific surface protein or component. To identify a possible hLF binding surface protein from *E. faecalis*, we coupled hLF to CnBr activated sepharose and incubated the sepharose with an *E. faecalis* cell extract. The proteins that were bound to hLF were eluted and analyzed via SDS-PAGE. We observed only one distinct band with a size of around 50 kDa and identified the protein via mass spectrometry as a surface associated enolase (EF1961) (Protein identification report: File S1). To verify the interaction between hLF and the enolase we immobilized recombinant enolase to a microtiter plate in an ELISA experiment to show binding of hLF to the enolase *in vitro* (Fig. 6 B).

Surface displayed enolase is in streptococci known to bind host proteins like plasminogen, fibrinogen and mucin [37]–[39], but to our knowledge, nothing is known about its interaction with hLF. Our results indicate that hLF inhibits biofilm formation by binding to the surface of *E. faecalis*, that this process is glycan dependent, and that the binding might be, at least in part, mediated through binding to the surface associated enolase. In addition, restoration of the biofilm formation by EndoE indicates that *E. faecalis* potentially uses this endoglycosidase to reverse the effect of biofilm inhibition by hLF.

### *E. faecalis* is able to Utilize the Glycans from Lactoferrin as Nutrients

**Figure 7:**
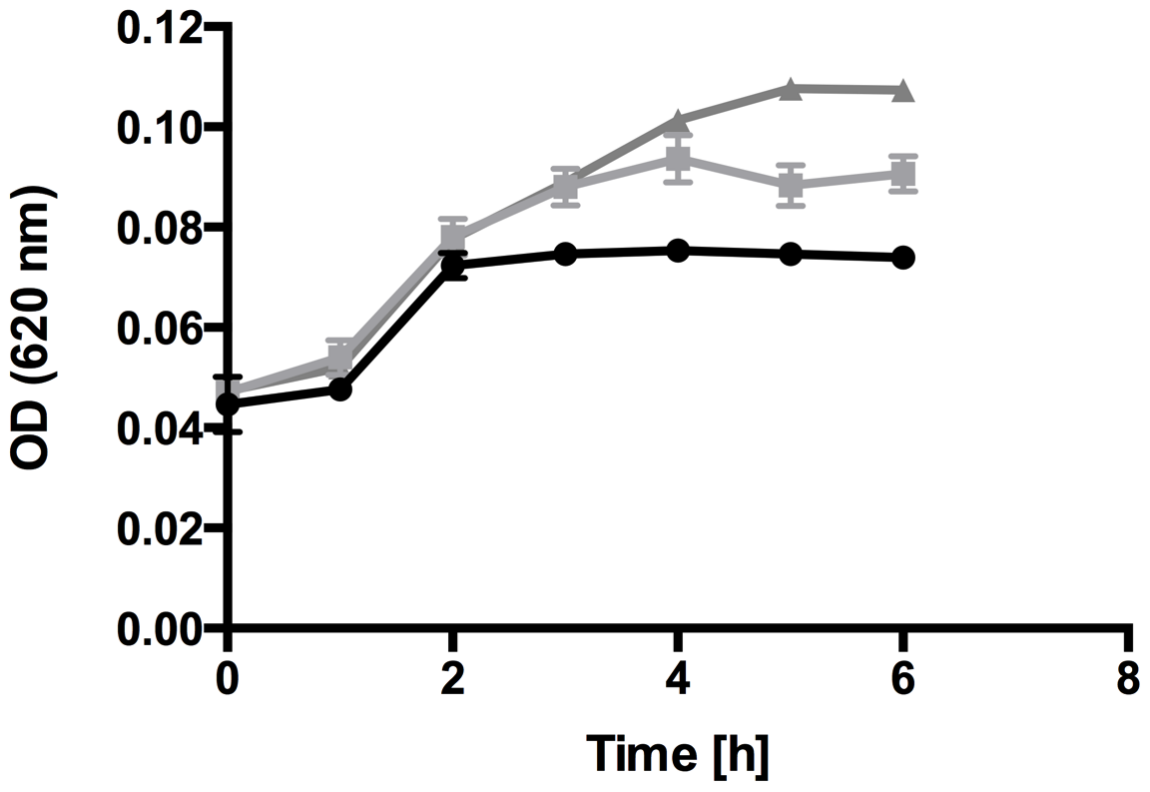
Growth of *Enterococcus faecalis* in the presence of lactoferrin. Growth curve of *E. faecalis* in diluted THB medium (•) and in diluted THB medium supplemented with 2 mg/ml hLF (▪) or isolated N-linked glycans from hLF (▴). Optical density (OD) at 620 nm was determined at indicated time points. Error bars indicate the standard deviation from the mean of three independent experiments.

It has been described that *E. faecalis* is able to grow with RNaseB as a single carbon source and it was assumed that deglycosylation of glycoproteins could be important for nutrient acquisition during growth *in vivo* [40]. Especially in nutrient poor environments, the glycans released from glycoproteins could be essential for the bacteria to persist and survive in a certain environment. Therefore, we were interested in whether *E. faecalis* is able to use the complex glycans from hLF to promote its growth. Culture experiments were performed in diluted THB medium with and without hLF as well as with the N-linked glycans isolated from hLF. This showed that *E. faecalis* is able to reach a higher optical density in the presence of hLF and in the presence of the isolated glycans than in diluted THB medium, indicating that the glycans can indeed promote the growth of *E. faecalis* (Fig. 7).

## Discussion

Glycosidases are widespread among bacterial pathogens and non-pathogens and play key roles in modulating host proteins, nutrient acquisition and escaping the immune system [14], [17], [18], [41]. There is limited information about the biological function of the endoglycosidase EndoE from the opportunistic pathogen *E. faecalis.* It cleaves the glycans from human IgG [22] and might be important to gain nutrients [40]. In this study we identified a new substrate for EndoE, the human glycoprotein lactoferrin (hLF). hLF is a 80 kDa non-heme iron binding protein considered to be a component of innate immunity [27], [42], [43]. Using lectin blot, UHPLC and LC-MS analysis, we showed that EndoE hydrolyzes glycans from hLF and that the activity of EndoE on hLF is dependent on the α-domain with GH18 activity. Moreover, we were able to show that most glycans released from hLF by EndoE correspond to the glycans released by PNGaseF. Due to the difference in site of cleavage of EndoE and PNGaseF, the glycoforms show up with a shift of roughly 0.5 GU (e.g. peak 6 and 15, as well as 10 and 21). Nevertheless, in the chromatogram with glycans released by EndoE, peaks 2, 4 and 5 represents glycans with single antennary structures (A1_E_G(4)1, A1_E_G1F(2)1, A1_E_G(4)1S(3)1) not present in the PNGaseF glycan profile. This phenomenon can be explained in two ways; first, PNGaseF does not have activity on these glycans and they are therefore not released from the glycoprotein. Or secondly, EndoE harbors a glycan hydrolyzing activity that further trims the glycans and gives rise to the observed peaks. Due to the broad activity of PNGaseF and the two active sites of EndoE we favor the second explanation. It can be speculated that EndoE holds two functions; one to modulate the function of the glycoprotein hLF by releasing the N-linked glycan and the other function to further process the released complex glycans. As judged from the lectin blot, it also seems like that the α-domain is responsible for the glycan release based on the glycosidase activity between the two GlcNAcs. The β-domain on the other hand could be responsible for the further trimming of the glycans by glycosidase activity between mannose and GlcNAc, which could not been seen with lectin blot analysis since the lectin ConA binds to mannose within the glycan. This is also supported by the similarity between EndoE and the exoglycosidase StrH from *Streptococcus pneumoniae* which also cleaves between mannose and GlcNAc [22], [44]. The absence of glycans that contain sialic acids α1–6 attached to galactose in the chromatogram obtained from EndoE could have two reasons: either EndoE has no activity on this type of glycans due to for example steric hindrance, or EndoE has a strong preference for non-sialylated glycans. In both cases the glycans would not appear in the chromatogram. However, independently of the incubation time of hLF with EndoE, fully glycosylated hLF was detected in the lectin blot analysis indicating that EndoE might not have activity on all hLF glycans. Taken together, EndoE hydrolyzes most glycoforms of hLF, and might even harbor two different glycosidase activities on hLF. Further investigation of the released glycans by the different domains of EndoE will help to explain the appearance of the glycans with single antennary structure and whether EndoE is able to cleave glycans with α1–6 linked sialic acids or not.

As an inhabitant of the gastrointestinal tract, but also as an opportunistic pathogen, *E. faecalis* encounters different niches where it is exposed to hLF. However, as described recently, hLF contains 17 structurally different glycans [35] and it is not known to what extent the glycans differ in the diverse niches within the human body. To our knowledge, the role of the N-linked glycans from hLF is poorly investigated with regards to the interaction with bacteria. hLF has been described to have antimicrobial activity, both bacteriostatic and bactericidal, and it has been shown to interact with bacteria in different ways. Bactericidal effects are explained by the inhibition of the bacterial H+-ATPase and with the direct interaction of hLF to bacterial surfaces that results in the damage of the outer membrane in Gram-negative bacteria, or the cell wall in Gram-positive bacteria [26], [31], [45], [46]. The bacteriostatic activity of hLF can be explained by iron deprivation of the bacteria [29], [47]. This iron sequestration was also suggested to be important for the biofilm inhibition of the Gram-negative bacterium *Pseudomonas aeruginosa* [32]. Biofilm inhibition by hLF has also been observed for other bacteria such as *Streptococcus mutans, Porphyromonas gingivalis* and *Prevotella intermedia* [33], [34]. However, the exact mechanism of biofilm inhibition is not clear yet and it is unclear whether the inhibition of biofilms by hLF is a universal effect or specific to certain bacteria. Biofilm-related infections are estimated to be responsible for up to 60% of nosocomial infections [48] and therefore play a critical role in the treatment of infections, since growth in a biofilm is often associated with a higher antibiotic resistance, which has also been described for enterococci [49]. Here we report a novel mechanism for the biofilm inhibition of *E. faecalis* by hLF. We showed in a crystal violet assay that hLF strongly inhibits biofilm formation. Deglycosylation of hLF by the endoglycosidase EndoE on the other hand partially restored the biofilm forming ability of *E. faecalis*, strongly suggesting that the inhibition is dependent on the glycans of hLF. Removal of glycans does not change the affinity to iron as reported previously [36] and a biofilm inhibitory effect due to iron sequestration can therefore be excluded. Moreover, we showed that hLF binds to the surface of *E. faecalis* and that this binding is also glycan dependent. A possible reason for the biofilm inhibition could be that the attachment or adhesion to surfaces is decreased due to binding of hLF to *E. faecalis.* It has also been described for *Actinobacillus actinomycetemcomitans* and *Prevotella intermedia* that hLF inhibits the adhesion to fibroblasts and epithelial cells suggesting that hLF prevents the adhesion of bacteria to periodontal tissues [50]. The activity of EndoE on the complex type glycans of hLF could be a mechanism for *E. faecalis* to reverse the biofilm inhibiting effect.

We here identified a surface associated hLF binding protein of *E. faecalis*, the α-enolase. This protein has until now not been known to interact with hLF. The enolase belongs to the so-called moonlighting enzymes with multifunctional properties. These proteins are often involved in metabolic processes, for example, the enolase converts 2-phosphoglycerate to phosphoenolpyruvate in the glycolytic pathway [51], [52]. On the other hand, as mentioned above, it has been shown that this protein is not only present intracellularly but interacts as a surface protein with other human proteins like plasminogen, fibrinogen and mucin [37]–[39]. It has been speculated that these additional biological functions are involved in bacterial virulence [51], [52]. Moreover, it is not known if this protein is important for the biofilm formation of *E. faecalis* or other bacteria. Future studies concerning the interaction of the enolase with hLF and its role in the biofilm formation of *E. faecalis* will most likely reveal additional details of the mechanism proposed above.

It has previously been described that the glycans of glycoproteins can serve as nutrients for bacteria and help to survive or persist *in vivo* [14], [21], [40]. We showed that the glycans of hLF can promote growth of *E. faecalis* in a nutrient limited medium. It seems likely that these glycans could also promote growth of cells within the nutrient limited environment of a biofilm. Similar to the enolase that is involved in metabolism and virulence, EndoE might also be important for metabolic purposes and virulence in *E. faecalis*.

We here provide novel information about the biofilm inhibiting potential of hLF and a possible function of its glycans when interacting with bacteria like *E. faecalis*. EndoE could have two roles: nutrient acquisition and to reverse the biofilm inhibiting effect of hLF. Taken together, the results of this study underline the importance of glycans in the interplay between bacteria and the human host, with possible implications for both commensalism and opportunism.

## Materials and Methods

### Bacterial Strains and Plasmids

The bacterial strains and plasmids used in this study are listed in Table 1. *E. coli* was routinely propagated in LB broth (Difco, Detroit, MI) at 37°C with aeration. *E. faecalis* strains were cultured in Todd-Hewitt broth (THB; Difco, Detroit, MI) at 37°C without aeration. 1.5% (w/v) agar (Difco, Detroit, MI) was used to solidify the medium when needed. Antibiotics were used at the following concentrations: carbenicillin 100 µg/ml (*E. coli*), chloramphenicol 34 µg/ml (*E. coli*).

**Table 1:** Bacterial strains and plasmids used in this study.

Strain or plasmid	Characteristics	Reference or source
***E. faecalis***		
OG1RF	Derivative of *E. faecalis* OG1, resistant to rifampicin	[57]
***E. coli***		
TOP10	Cloning strain	Invitrogen
BL21(DE3)pLys	Cloning/expression strain	Invitrogen
**Plasmids**		
pCR2.1	PCR cloning vector	Invitrogen
pGEX-5X-3	GST fusion vector	GE Healthcare
pGEX*ndoE*	Expression vector for full length EndoE	[22]
pGEX*ndoE*(E186Q)	E186Q mutation in EndoE	[22]
pGEX*ndoE*(E662Q)	E662Q mutation in EndoE	[22]
pGEX EF1961	Expression vector for full length EF1961	This study

### Growth Experiments with Lactoferrin

50 µg human lactoferrin from milk (hLF) (Sigma-Aldrich) was incubated with 50 µg recombinant EndoE in PBS at 37°C overnight and released glycans were separated from the mixture using 10 MWCO centrifugal filters. *E. faecalis* was cultured overnight in THB and then diluted 1∶20 in 20× diluted THB medium with addition of 2 mg/mL hLF or released glycans from approximately 50 µg hLF. The growth was followed by measuring OD at 620 nm over time.

### PCR Analysis and Sequencing

To amplify DNA from *E. faecalis* strains, genomic DNA was isolated using the DNA extraction kit Gentra Puregene Yeast/Bact (Qiagen AB, Sweden) according to the manufacturer’s instructions. 30 ng genomic DNA was used as a template in a standard PCR using TrueStart *Taq* polymerase (Fermentas AB, Sweden). PCR products were separated on 1% TAE agarose and stained with SYBR Safe (Invitrogen). PCR fragments or plasmids were sequenced using the sequencing service of GATC Biotech (Konstanz, Germany).

### Recombinant Expression of EndoE

EndoE, EndoE(E186Q) and EndoE(E662Q) were recombinantly expressed in *E. coli* as previously described using the GST gene fusion system from GE Healthcare [22]. Recombinant expression of the enolase (EF1961) was performed using the same protocol. Briefly, a 1299 bp fragment of EF1961 was amplified using the following primers: 5′-CGGGATCCACATGTCAATTATTACTGATA-3′ with a *BamH*I site (underlined) and 5′-CCCTCGAGTTATTTGTTTTTTAAGTTG-3′ with an *Xho*I site (underlined). The PCR fragment was cloned into the pGEX-5x-3 vector (GE Healthcare) and transformed into the *E. coli* BL21 (DE3) pLysE (Invitrogen) strain for expression of EF1961.

### Lectin Blot Analysis

Activity of EndoE on human lactoferrin from milk (hLF) (Sigma-Aldrich) was tested as described with other glycoproteins previously [22]. Briefly, 2 µg of hLF was incubated with 2 µg recombinant EndoE, EndoE(E186Q) or EndoE(E662Q) in a total volume of 20 µl of PBS buffer for 16 h at 37°C. Activity was analyzed with 10% SDS PAGE (stained with Coomassie Brilliant Blue G-250) and lectin blot analysis was performed using 0.5 µg/ml biotinylated ConA lectin (Vector Laboratories, Burlingame, CA) [22], [53].

### UHPLC Analysis

Glycans from 1 µg hLF were released with EndoE or N-glycosidase F (PNGaseF; ProZyme) in PBS at 37°C overnight. The released glycans were isolated with 10 MWCO spin columns (Pall), desalted using porous graphitic carbon and labeled with 2-AB using the LudgerTag Glycan Labeling Kit (Ludger Ltd) according to the manufacturer’s instructions. Glycans labeled with 2-AB were analyzed using a Waters 1.7 µm BEH Glycan (2.1 mm×150 mm) column coupled to a Waters H-class UHPLC instrument. Separations were carried out using a 30–58% gradient of 50 mM ammonium formate pH 4.4 against acetonitrile with a column temperature of 40°C over a 30 min duration. Labeled glycans were visualized using fluorescence detection with excitation and emission wavelengths of 330 nm and 420 nm, respectively. Individual peaks appearing within chromatograms were converted to glucose unit (GU) values for comparative purposes. GU values were calculated through use of a 2-AB labeled dextran hydrolysate as a glycan ladder, which provided a means of converting peak retention time to a standardized GU value [54]. Retention times for peaks of interest in each glycan sample were integrated and converted to GU values using Empower 3 software (Waters, MA, USA).

### LC-FLD-MS

Online coupled fluorescence (FLD)-mass spectrometry detection was performed using a Waters Xevo G2 QTof with Acquity UPLC and BEH Glycan column (1.0×150 mm, 1.7 µm particle size). MS data was acquired in negative mode with the following conditions: 2500 V capillary voltage, 50 V cone voltage, 280°C desolvation temperature, 600 L/hour desolvation gas and 100°C source temperature. The analyzer was set to sensitivity mode. The fluorescence data rate was 1 pts/second and a PMT gain = 10 with altering excitation and emission wavelengths that varied based on the experiment and glycan label used. Sample injection volumes were 10 µl and the flow rate was 0.150 µl/min. Solvent A was 50 mM ammonium formate pH 4.4 and solvent B was acetonitrile. A 40 minute linear gradient was used and was as follows: 28–43% A for 32 minutes, 70% A for 4 minutes and 28% solvent A for 4 minutes. Samples were diluted in 65% acetonitrile prior to analysis.

### BIAcore Surface Plasmon Resonance (SPR) Interaction Analysis

Lactoferrin was diluted in 10 mM sodium acetate, pH 4, and immobilized via amine coupling to a flow cell of Sensor Chip CM3 (GE Healthcare, Uppsala, Sweden). Immobilization levels were optimized to around 1000 response units. A flow cell subjected to the immobilization protocol but without any addition of protein was used as control for buffer bulk changes and nonspecificity.

For affinity measurements, the binding and dissociation phases were monitored with a BIAcore 2000 instrument (GE Healthcare). In control experiments for possible mass transfer limitations, EndoE variants were injected over the surfaces at different flow rates. No differences in initial binding were observed at 5 µl/min or above indicating no limitations to any combinations of ligands. EndoE(E186Q) and EndoE(E662Q) were injected at different concentrations (typically 31–500 nM) at 15 µl/min and 25°C over the lactoferrin flow cell in running buffer (10 mM Hepes, pH 7.5, 150 mM NaCl, and 0.005% surfactant P20).

Between experiments, the surfaces were strictly regenerated with micro-pulses of 0.1 M NaHCO_3_, pH 12 and with running buffer containing 2 M NaCl followed by an extensive wash procedure after reaching flow cell baseline. After X and Y normalization of data (injection starting points set to zero), the blank curves from control flow cells of each injected concentration were subtracted.

The association (k_a_) and dissociation (k_d_) rate constants were determined simultaneously using the equation for 1∶1 Langmuir binding in the BIA Evaluation 4.1 software (GE Healthcare). The binding curves were fitted locally and the equilibration dissociation constants (K_D_) were calculated from mean values of the obtained rate constants.

### Biofilm Assay

Quantification of biofilm formation was performed using a modified crystal violet microtiter plate assay as described previously [55]. A bacterial overnight culture was diluted 1∶10 in THB medium supplemented with 0.4% glucose, 200 µl of bacterial suspension was filled in each flat bottomed 96-well microtiter plate (Nunc) and incubated for 24 h at 37°C, 5% CO_2_. The content of each well was discarded and the wells were washed three times with 190 µl sterile PBS buffer. The attached bacteria were fixed with 200 µl methanol for 10 min. The methanol was discarded and the wells air-dried at room temperature for 15 min. Bacteria were stained for 5 min with 160 µl 1% (w/v in H_2_O) crystal violet solution. After staining, the wells were washed three times with 190 µl PBS and the bound dye was finally re-solubilized with 200 µl ethanol/acetone plate and the OD was immediately measured at a wavelength of 550 nm. To study the biofilm formation in the presence of lactoferrin the protein was directly added to the diluted bacteria at indicated concentrations. For deglycosylation, hLF was first incubated with recombinant EndoE-GST fusion protein in equal amounts for 16 h at 37°C. EndoE was removed using glutathione sepharose (GE Healthcare) and the EndoE treated hLF (de-hLF) was sterile filtered. Each experiment is displayed as the mean of three independent experiments with three replicates.

To study the amount of viable cells in the biofilm assay, control cultures were vigorously mixed after 24 h to detach the cells from the bottom of the well, diluted in PBS-buffer and the colony forming unit (CFU) was determined. Each experiment was performed in three independent experiments.

### Binding of Lactoferrin to *Enterococcus faecalis*

5 ml stationary phase culture of *E. faecalis* was washed 3 times with PBS buffer and resuspended in PBS buffer. 50 µl cells (2.5×10^7^ cells) were incubated with indicated concentrations of human lactoferrin (hLF) or EndoE treated hLF (de-hLF, see above) for 2 h at 37°C. The bacteria were washed 3 times with PBS to remove non-bound hLF, pelleted and dissolved in SDS-buffer. Cell extract was separated on 10% SDS-PAGE and electro-blotted onto PVDF membranes. Membranes were processed as described [22] and hLF was detected using anti-lactoferrin antibodies.

### Identification of a Lactoferrin Binding Protein from *Enterococcus faecalis*

In order to identify a protein on the enterococcal surface that binds lactoferrin, lactoferrin was coupled to CnBr-activated sepharose (Pharmacia Biotech) according to the manufacturer’s instructions. 30 ml of enterococcal over night culture was washed twice with PBS buffer, resuspended in 10 ml of 0.01 M KH_2_PO_4_ buffer, pH 6.2 and treated with 250 U mutanolysin at 37°C until lysis occurred. The lysate was centrifuged to remove cell debris and the supernatant was incubated with the CnBr activated sepharose with coupled lactoferrin. The lactoferrin bound protein from the enterococcal cell extract was eluted with 0.1 M glycine, pH 2 and concentrated using trichloroacetic acid at a final concentration of 5%. Proteins were separated on 10% SDS-PAGE, stained with Coomassie Brilliant Blue R-250 and identified using the protein identification service from Alphalyse (Odense, Denmark).

### Enzyme Linked Immunosorbent Assay (ELISA)

Microtiter plates (MaxiSorp, NUNC, Roskilde, Denmark) were coated with 5 µg/ml hLF in coatingbuffer (16 mM Na_2_CO_3_ and 35 mM NaHCO_3_, pH 9.6) at 4°C overnight. The plates were washed three times with PBS +0.05% Tween (PBST). Non-specific binding was blocked with 2% (w/v) BSA in PBST for 30 min at room temperature followed by washing with PBST. hLF was diluted in PBST +2% (w/v) BSA (concentrations indicated), added to the wells and incubated for 1 h at 37°C. Wells were washed three times with PBST, anti-hLF antibody was added (1∶1000 in PBST +2% (w/v) BSA) and incubation proceeded for another hour at 37°C. Secondary HRP conjugated antibody (1∶3000 in PBST +2% (w/v) BSA) was added after washing three times with PBST. Following three more washes, the color reaction was developed with 0.1 M citric acid monohydrate, 0.1 M Na_2_HPO_4_×2H_2_O buffer pH 4.5 containing 0.012% (v/v) H_2_O_2_ and 1.7 mM 2,29-azino-bis(3- ethylbenzthiazoline-6-sulphonic acid) (ABTS). The absorbance was read on a model 550 micro plate reader (BIO-RAD, Hercules, CA, USA) at 420 nm. The experiments were made in triplicates.

## Supporting Information

File S1 Protein identification report and MS spectrum.
(PDF)
