# Supplementary material for: EndoE from Enterococcus faecalis Hydrolyzes the Glycans of the Biofilm Inhibiting Protein Lactoferrin and Mediates Growth
Source: PLoS One. 2014 Mar 7;9(3):e91035. doi: 10.1371/journal.pone.0091035 (PMC3946673; doi:10.1371/journal.pone.0091035)
Supplement: File S1 — Protein identification report and MS spectrum. (PDF) [file pone.0091035.s001.pdf]

Protein Identification Report

Order 13792

Julia Garbe

Lund Universitet

Date: October 04, 2012

Protein Identification Report

Order 13792

Overview

Mass spectrometric peptide mapping and sequencing analysis

Customer samples:

Sample name	Protein found in database	GI-number	MW	Score	Seq. cov.	Note
1	phosphopyruvate hydratase [Enterococcus faecalis TX1467]	gi|329568718	46454	502	55%	

Quality control standards included in the analysis

Standard	Protein found in database	GI-number	MW	Score	Seq. cov.	Note
1 pmol BSA	Chain A, Crystal Structure Of Bovine Serum Albumin	gi|367460260	68416	941	38%	
62 fmol Transferrin	Chain A, Apo-Human Serum Transferrin (Glycosylated)	gi|110590599	76988	198	28%	

Alphalyse A/S ■ Unsbjergvej 4 ■ DK-5220 Odense ■ Denmark
Tel: +45 6310 6500 ■ e-mail info@alphalyse.com
www.alphalyse.com

Experimental

Samples received

The following sample was received at Alphalyse for protein analysis.

1

Protein identification by MS peptide mapping and sequencing analysis

The protein samples were reduced and alkylated with iodoacetamide, i.e. carbamidomethylated, and subsequently digested with trypsin that cleaves after lysine and arginine residues. The resulting peptides were concentrated on a ZipTip micropurification column and eluted onto an anchorchip target for analysis on a Bruker Autoflex III MALDI TOF/TOF instrument. The peptide mixture was analyzed in positive reflector mode for accurate peptide mass determination. MALDI MS/MS was performed on 15 peptides for peptide fragmentation analysis, i.e partial sequencing. The MS and MS/MS spectra were combined and used for database searching using the Mascot software. The data are searched against in-house protein databases downloaded from NCBI, including the NRDB database containing more than 15 million known non-redundant protein sequences. The data can also be searched against a custom database containing specific protein sequences provided by the client.

The Mascot software finds matching proteins in the database by their peptide masses and peptide fragment masses. The protein identification is based on a probability-scoring algorithm (www.matrixscience.com) and the significant best matching protein is shown in the result report. Homologous proteins with a lower score are not included in the report. If the protein from the correct organism is not present in the database, then a significant matching homologous protein from another organism is reported. If several proteins are identified with a significant score then several protein identifications are reported for the sample.

The identified database protein sequences are shown in the Results together with the obtained mass spectrometric peptide maps. The peptides used for the identification are highlighted in the sequence and the matching peptides are listed for comparison of the determined and calculated values. Peptide identities that are confirmed by MS/MS fragment analysis are shown in bold with an Ions Score.

Bioinformatics tools

Bioinformatics Tools are show as hyperlinks for each identified protein in the report. The NRDB database entry for the identified protein can be looked up at the NCBI website (National Center for Biotechnology Information) by selecting the NCBI Entry hyperlink. A Blast homology search in the NCBI database is found by selecting Blink NCBI. Known functional domains in the protein can be found in the Conserved Domain Database (Conserved Domains in NCBI).

The Bioinformatics Guide at (www.alphalyse.com/bioinformatics-guide.html) explains and guides you through a range of important bioinformatics tools to let you investigate the function and properties of the protein. The guide contains hyperlinks to bioinformatics search forms and case examples on how the tools are used.

Alphalyse A/S ■ Unsbjergvej 4 ■ DK-5220 Odense ■ Denmark
Tel: +45 6310 6500 ■ e-mail info@alphalyse.com
www.alphalyse.com

Results

Sample name: 1

Protein Information

**Protein name:**	**phosphopyruvate hydratase [Enterococcus faecalis TX1467]**
Alphalyse number:	ALPHA24416
GI-number:	gi|329568718
MW:	46454
pI:	4,58
Mascot score:	502
Sequence coverage:	55%
Bioinformatic tools:	1: NCBI Entry 2: Blink (Blast) NCBI 3: Conserved Domains in NCBI

Analysis Information

- MS analysis method: MALDI-TOF peptide mass fingerprint and MALDI-TOF/TOF peptide sequencing
- Enzyme: Trypsin
- Variable modifications: Oxidation (M)
- Fixed modifications: Carbamidomethyl (C)
- Database search program: Mascot version 2.2.03
- Peptide Tolerance: 60 ppm
- Allowed up to 1 miscleavage
- Database: NRDB1 (17378729 protein sequences)

Protein sequence

Matched peptides shown in bold underline

```
  1 MSIITDIYAR EVLDSRGNPT IEVEVYTESG AFGRGMVPSG ASTGEYEAVE
 51 LRDGDKARYL GKGVTKAVDN VNNIIAEAII GYDVRDQMAI DKAMIDLDGT
101 PNKGKLGANA ILGVSIAVAR AAADYLEVPL YHYLGGFNTK VLPTPMMNII
151 NGGSHADNSI DFQEFMIMPV GAPTFKEALR MGAEVFHALA SILKGRGLAT
201 SVGDEGGFAP NLGSNEEGFE VIIEAIEKAG YVPGKDVVLA MDAASSEFYD
251 KEKGVYVLAD SGEGEKTTEE MIAFYEELVS KYPIISIEDG LDENNWDGFK
301 KLTEVLGDKV QLVGDDLFVT NTTKLAEGIE KGIANSILIK VSQIGTLTET
351 FEAIEMAKEA GYTAVVSHRS GETEDSTISD IAVATNAGQI KTGSLSRTDR
401 IAKYNQLLRI EDQLGDVAEY KGLKSFYNLK NK
```

Peptides used for identification

Peptides shown in bold have been analysed by MS/MS sequencing

Start - End	Observed	Mr(expt)	Mr(calc)	Delta	Miss	Sequence
**2 - 10**	**1051.54**	**1050.54**	**1050.57**	**-33.00**	**0**	**M.SIITDIYAR.E (Ions score 49)**
**17 - 34**	**1925.90**	**1924.89**	**1924.92**	**-13.00**	**0**	**R.GNPTIEVEVYTESGAFGR.G (Ions score 128)**
**35 - 52**	**1852.84**	**1851.83**	**1851.87**	**-20.00**	**0**	**R.GMVPSGASTGEYEAVELR.D (Ions score 34)**
35 - 52	1868.84	1867.84	1867.86	-14.00	0	R.GMVPSGASTGEYEAVELR.D Oxidation (M)
35 - 56	2268.04	2267.04	2267.04	-1.00	1	R.GMVPSGASTGEYEAVELRDGDK.A
**67 - 85**	**2059.05**	**2058.04**	**2058.07**	**-16.00**	**0**	**K.AVDNVNNIIAEAIIGYDVR.D (Ions score 42)**
93 - 105	1359.69	1358.68	1358.69	-5.00	1	K.AMIDLDGTPNKGK.L
**106 - 120**	**1424.83**	**1423.82**	**1423.85**	**-20.00**	**0**	**K.LGANAILGVSIAVAR.A (Ions score 1)**
121 - 140	2242.08	2241.08	2241.11	-16.00	0	R.AAADYLEVPLYHYLGGFNTK.V

Alphalyse A/S ■ Unsbjergvej 4 ■ DK-5220 Odense ■ Denmark
Tel: +45 6310 6500 ■ e-mail info@alphalyse.com
www.alphalyse.com

181 - 194	1486.77	1485.76	1485.80	-28.00	0	R.MGAEVFHALASILK.G
181 - 194	1502.76	1501.75	1501.80	-30.00	0	R.MGAEVFHALASILK.G Oxidation (M)
282 - 300	2225.07	2224.06	2224.03	13.00	0	K.YPIISIEDGLDENNWDGFK.K
302 - 324	2505.27	2504.26	2504.34	-31.00	1	K.LTEVLGDKVQLVGDDLFVTNTTK.L
**332 - 340**	**928.54**	**927.54**	**927.58**	**-42.00**	**0**	**K.GIANSILIK.V (Ions score 9)**
**359 - 369**	**1189.57**	**1188.56**	**1188.59**	**-25.00**	**0**	**K.EAGYTAVVSHR.S (Ions score 84)**
370 - 391	2207.02	2206.02	2206.06	-20.00	0	R.SGETEDSTISDIAVATNAGQIK.T
404 - 409	806.41	805.40	805.44	-53.00	0	K.YNQLLR.I
**410 - 421**	**1379.65**	**1378.64**	**1378.66**	**-15.00**	**0**	**R.IEDQLGDVAEYK.G (Ions score 9)**
410 - 424	1677.84	1676.83	1676.86	-17.00	1	R.IEDQLGDVAEYKGLK.S
425 - 430	771.36	770.35	770.40	-56.00	0	K.SFYNLK.N

Alphalyse A/S ■ Unsbjergvej 4 ■ DK-5220 Odense ■ Denmark
Tel: +45 6310 6500 ■ e-mail info@alphalyse.com
www.alphalyse.com

Quality control standards

Sample name: 1 pmol BSA

Protein Information

Protein name:	Chain A, Crystal Structure Of Bovine Serum Albumin
Alphalyse number:	Standard1
GI-number:	gi|367460260
MW:	68416
pI:	5,6
Mascot score:	941
Sequence coverage:	38%
Bioinformatic tools:	1: NCBI Entry 2: Blink (Blast) NCBI 3: Conserved Domains in NCBI

Analysis Information

- MS analysis method: MALDI-TOF peptide mass fingerprint and MALDI-TOF/TOF peptide sequencing
- Enzyme: Trypsin
- Variable modifications: Oxidation (M)
- Fixed modifications: Carbamidomethyl (C)
- Database search program: Mascot version 2.2.03
- Peptide Tolerance: 60 ppm
- Allowed up to 1 miscleavage
- Database: NRDB1 (17378729 protein sequences)

Protein sequence

Matched peptides shown in bold underline

```
  1 DTHKSEIAHR FKDLGEEHFK GLVLIAFSQY LQQCPFDEHV KLVNELTEFA
 51 KTCVADESHA GCEKSLHTLF GDELCKVASL RETYGDMADC CEKQEPERNE
101 CFLSHKDDSP DLPKLKPDPN TLCDEFKADE KKFWGKYLYE IARRHPYFYA
151 PELLYYANKY NGVFQECCQA EDKGACLLPK IETMREKVLT SSARQRLRCA
201 SIQKFGERAL KAWSVARLSQ KFPKAEFVEV TKLVTDLTKV HKECCHGDLL
251 ECADDRADLA KYICDNQDTI SSKLKECCDK PLLEKSHCIA EVEKDAIPEN
301 LPPLTADFAE DKDVCKNYQE AKDAFLGSFL YEYSRRHPEY AVSVLLRLAK
351 EYEATLEECC AKDDPHACYS TVFDKLKHLV DEPQNLIKQN CDQFEKLGEY
401 GFQNALIVRY TRKVPQVSTP TLVEVSRSLG KVGTRCCTKP ESERMPCTED
451 YLSLILNRLC VLHEKTPVSE KVTKCCTESL VNRRPCFSAL TPDETYVPKA
501 FDEKLFTFHA DICTLPDTEK QIKKQTALVE LLKHKPKATE EQLKTVMENF
551 VAFVDKCCAA DDKEACFAVE GPKLVVSTQT ALA
```

Peptides used for identification

Peptides shown in bold have been analysed by MS/MS sequencing

Start - End	Observed	Mr(expt)	Mr(calc)	Delta	Miss	Sequence
**11 - 20**	**1249.57**	**1248.56**	**1248.61**	**-40.00**	**1**	**R.FKDLGEEHFK.G (Ions score 55)**
42 - 51	1163.61	1162.60	1162.62	-18.00	0	K.LVNELTEFAK.T
65 - 76	1419.64	1418.63	1418.69	-39.00	0	K.SLHTLFGDELCK.V
115 - 131	2019.90	2018.89	2018.96	-34.00	1	K.LKPDPNTLCDEFKADEK.K
**137 - 143**	**927.46**	**926.45**	**926.49**	**-37.00**	**0**	**K.YLYEIAR.R (Ions score 48)**
**137 - 144**	**1083.56**	**1082.55**	**1082.59**	**-36.00**	**1**	**K.YLYEIARR.H (Ions score 36)**

Alphalyse A/S ■ Unsbjergvej 4 ■ DK-5220 Odense ■ Denmark
Tel: +45 6310 6500 ■ e-mail info@alphalyse.com
www.alphalyse.com

**144 - 159**	**2044.99**	**2043.99**	**2044.02**	**-17.00**	**1**	**R.RHPYFYAPELLYYANK.Y (Ions score 68)**
209 - 217	1001.54	1000.54	1000.58	-44.00	1	R.ALKAWSVAR.L
274 - 285	1532.72	1531.71	1531.77	-40.00	1	K.LKECCDKPLLEK.S
317 - 335	2301.10	2300.09	2300.07	7.00	1	K.NYQEAKDAFLGSFLYEYSR.R
**323 - 335**	**1567.71**	**1566.70**	**1566.74**	**-20.00**	**0**	**K.DAFLGSFLYEYSR.R (Ions score 109)**
**336 - 347**	**1439.77**	**1438.76**	**1438.80**	**-32.00**	**1**	**R.RHPEYAVSVLLR.L (Ions score 73)**
337 - 347	1283.66	1282.65	1282.70	-38.00	0	R.HPEYAVSVLLR.L
378 - 388	1305.66	1304.65	1304.71	-42.00	0	K.HLVDEPQNLIK.Q
389 - 409	2529.18	2528.17	2528.21	-16.00	1	K.QNCDQFEKLGEYGFQNALIVR.Y
**397 - 409**	**1479.76**	**1478.75**	**1478.79**	**-26.00**	**0**	**K.LGEYGFQNALIVR.Y (Ions score 108)**
**413 - 427**	**1639.89**	**1638.89**	**1638.93**	**-27.00**	**1**	**R.KVPQVSTPTLVEVSR.S (Ions score 137)**
414 - 427	1511.79	1510.78	1510.84	-35.00	0	K.VPQVSTPTLVEVSR.S
445 - 458	1724.80	1723.79	1723.83	-22.00	0	R.MPCTEDYLSLILNR.L
**445 - 458**	**1740.79**	**1739.78**	**1739.82**	**-25.00**	**0**	**R.MPCTEDYLSLILNR.L Oxidation (M) (Ions score 72)**
459 - 471	1539.76	1538.75	1538.81	-39.00	1	R.LCVLHEKTPVSEK.V
475 - 499	3000.36	2999.35	2999.39	-14.00	1	K.CCTESLVNRRPCFSALTPDETYVPK.A
**484 - 499**	**1880.88**	**1879.87**	**1879.91**	**-22.00**	**0**	**R.RPCFSALTPDETYVPK.A (Ions score 81)**

Alphalyse A/S ■ Unsbjergvej 4 ■ DK-5220 Odense ■ Denmark
Tel: +45 6310 6500 ■ e-mail info@alphalyse.com
www.alphalyse.com

Protein Identification Report

Order 13792

Sample name: 62 fmol Transferrin

Protein Information

Protein name:	Chain A, Apo-Human Serum Transferrin (Glycosylated)
Alphalyse number:	Standard2
GI-number:	gi|110590599
MW:	76988
pI:	6,85
Mascot score:	198
Sequence coverage:	28%
Bioinformatic tools:	1: NCBI Entry 2: Blink (Blast) NCBI 3: Conserved Domains in NCBI

Analysis Information

- MS analysis method: MALDI-TOF peptide mass fingerprint and MALDI-TOF/TOF peptide sequencing
- Enzyme: Trypsin
- Variable modifications: Oxidation (M)
- Fixed modifications: Carbamidomethyl (C)
- Database search program: Mascot version 2.2.03
- Peptide Tolerance: 60 ppm
- Allowed up to 1 miscleavage
- Database: NRDB1 (17378729 protein sequences)

Protein sequence

Matched peptides shown in bold underline

1 KTVRWCAVSE HEATKCQSFR DHMKSVIPSD GPSVACVK**KA SYLDCIR**AIA
51 ANEADAVTLD AGLVYDAYLA PNNLKPVVAE FYGSKEDPQT FYYAVAVVK**K**
101 **DSGFQMNQLR** GKKSCHTGLG RSAGWNIPIG LLYCDLPEPR KPLEKAVANF
151 FSGSCAPCAD GTDFPQLCQL CPGCGCSTLN QYFGYSGAFK CLK**DGAGDVA**
201 **FVKHSTIFEN LANK**ADR**DQY ELLCLDNTRK PVDEYKDCHL AQVPSHTVVA**
251 **R**SMGGKEDLI WELLNQAQEH FGKDKSKEFQ LFSSPHGKDL LFKDSAHGFL
301 K**VPPRMDAKM YLGYEYVTAI R**NLREGTCPE APTDECKPVK **WCALSHHER**L
351 KCDEWSVNSV GKIECVSAET TEDCIAKIMN GEADAMSLDG GFVYIAGKCG
401 LVPVLAENYN KSDNCEDTPE AGYFAVAVVK KSASDLTWDN LKGKKSCHTA
451 VGRTAGWNIP MGLLYNK**INH CRFDEFFSEG CAPGSK**KDSS LCKLCMGSGL
501 NLCEPNNK**EG YYGYTGAFR**C LVEKGDVAFV KHQTVPQNTG GK**NPDPWAKN**
551 **LNEKDYELLC LDGTRKPVEE YANCHLAR**AP NHAVVTRKDK EACVHKILRQ
601 QQHLFGSNVT DCSGNFCLFR SETK**DLLFRD DTVCLAK**LHD RNTYEKYLGE
651 EYVKAVGNLR K**CSTSSLLEA CTFR**RP

Peptides used for identification

Peptides shown in bold have been analysed by MS/MS sequencing

Start - End	Observed	Mr(expt)	Mr(calc)	Delta	Miss	Sequence
39 - 47	1125.53	1124.52	1124.56	-38.00	1	K.KASYLDCIR.A
40 - 47	997.43	996.42	996.47	-49.00	0	K.ASYLDCIR.A
100 - 110	1323.66	1322.65	1322.64	11.00	1	K.KDSGFQMNQLR.G
101 - 110	1211.51	1210.51	1210.54	-29.00	0	K.DSGFQMNQLR.G Oxidation (M)
194 - 214	2233.06	2232.05	2232.12	-29.00	1	K.DGAGDVAFVKHSTIFENLANK.A
218 - 236	2399.02	2398.01	2398.15	-57.00	1	R.DQYELLCLDNTRKPVDEYK.D
230 - 236	878.47	877.47	877.45	12.00	0	R.KPVDEYK.D

Alphalyse A/S ■ Unsbjergvej 4 ■ DK-5220 Odense ■ Denmark
Tel: +45 6310 6500 ■ e-mail info@alphalyse.com
www.alphalyse.com

230 - 251	2549.26	2548.25	2548.29	-13.00	1	R.KPVDEYKDCHLAQVPSHTVVAR.S
302 - 309	913.49	912.49	912.49	0.00	1	K.VPPRMDAK.M
**310 - 321**	**1478.71**	**1477.70**	**1477.73**	**-20.00**	**0**	**K.MYLGYEYVTAIR.N (Ions score 1)**
**310 - 321**	**1494.69**	**1493.68**	**1493.72**	**-29.00**	**0**	**K.MYLGYEYVTAIR.N Oxidation (M) (Ions score 13)**
**341 - 349**	**1195.52**	**1194.51**	**1194.54**	**-20.00**	**0**	**K.WCALSHHER.L (Ions score 1)**
468 - 486	2257.98	2256.98	2256.97	4.00	1	K.INHCRFDEFFSEGCAPGSK.K
**509 - 519**	**1283.54**	**1282.53**	**1282.56**	**-23.00**	**0**	**K.EGYYGYTGAFR.C (Ions score 47)**
543 - 549	827.44	826.43	826.40	42.00	0	K.NPDPWAK.N
550 - 565	1952.89	1951.88	1951.93	-26.00	1	K.NLNEKDYELLCLDGTR.K
**566 - 578**	**1586.73**	**1585.73**	**1585.77**	**-26.00**	**0**	**R.KPVEEYANCHLAR.A (Ions score 24)**
**625 - 637**	**1565.76**	**1564.75**	**1564.79**	**-27.00**	**1**	**K.DLLFRDDTVCLAK.L (Ions score 14)**
**662 - 674**	**1531.65**	**1530.64**	**1530.68**	**-24.00**	**0**	**K.CSTSSLLEACTFR.R (Ions score 24)**

Alphalyse A/S ■ Unsbjergvej 4 ■ DK-5220 Odense ■ Denmark
Tel: +45 6310 6500 ■ e-mail info@alphalyse.com
www.alphalyse.com

MS spectrum

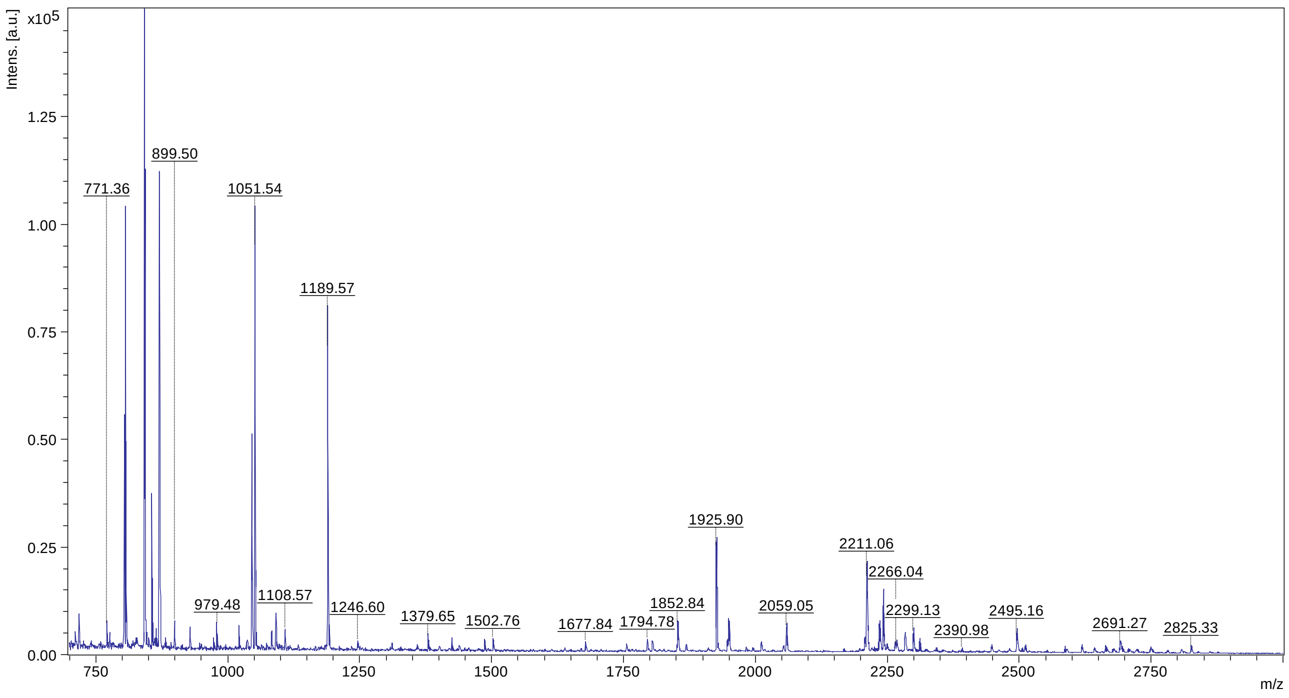

Alphalyse A/S ■ Unsbjergvej 4 ■ DK-5220 Odense ■ Denmark
Tel: +45 6310 6500 ■ e-mail info@alphalyse.com
www.alphalyse.com
